# afterParty: turning raw transcriptomes into permanent resources

**DOI:** 10.1186/1471-2105-14-301

**Published:** 2013-10-07

**Authors:** Martin Jones, Mark Blaxter

**Affiliations:** 1 Institute of Evolutionary Biology, University of Edinburgh, Edinburgh EH9 3JT, UK

**Keywords:** Transcriptome, Assembly, Annotation

## Abstract

**Background:**

Next-generation DNA sequencing technologies have made it possible to generate transcriptome data for novel organisms quickly and cheaply, to the extent that the effort required to annotate and publish a new transcriptome is greater than the effort required to sequence it. Often, following publication, details of the annotation effort are only available in summary form, hindering subsequent exploitation of the data. To promote best-practice in annotation and to ensure that data remain accessible, we have written afterParty, a web application that allows users to assemble, annotate and publish novel transcriptomes using only a web browser.

**Results:**

afterParty is a robust web application that implements best-practice transcriptome assembly, annotation, browsing, searching, and visualization. Users can turn a collection of reads (from Roche 454 chemistry) or assembled contigs (from any sequencing chemistry, including Illumina Solexa RNA-Seq) into a searchable, browsable transcriptome resource and quickly make it publicly available. Contigs are functionally annotated based on similarity to known sequences and protein domains. Once assembled and annotated, transcriptomes derived from multiple species or libraries can be compared and searched. afterParty datasets can either be created using the existing afterParty server, or using local instances that can be built easily using a virtual machine. afterParty includes powerful visualization tools for transcriptome dataset exploration and uses a flexible annotation architecture which will allow additional types of annotation to be added in the future.

**Conclusions:**

afterParty's main use case scenario is one in which a working biologist has generated a large volume of transcribed sequence data and wishes to turn it into a useful resource that has some durability. By reducing the effort, bioinformatics skills, and computational resources needed to annotate and publish a transcriptome, afterParty will facilitate the annotation and sharing of sequence data that would otherwise remain unavailable. A typical metazoan transcriptome containing several tens of thousands of contigs can be annotated in a few minutes of interactive time and a few days of computational time.

## Background

### Transcriptome sequencing

Recent advances in DNA sequencing technology have greatly reduced the cost, time and effort required to generate large volumes of sequence data [1]. While new sequencing approaches have been used to great effect in well-studied species [2,3], perhaps the biggest beneficiaries have been research programmes focussing on non-model organisms. For such organisms, which typically lack a reference genome sequence, transcriptome sequencing offers an efficient way to explore the regions of the genome likely to be of most interest to researchers [4,5]. The production of a novel transcriptome typically involves several steps [6]. mRNA is extracted from the organism of interest, purified, fragmented and reverse transcribed into cDNA. Several such cDNA collections may be made in order to capture transcripts that are only produced in specific tissue types, life stages, environmental conditions, etc. The cDNA molecules are then ligated to sequencing adapters and size-selected before having one or both ends sequenced. The result is a very large number of short reads that must undergo significant processing before they can be used to investigate the biology of the organism.

The details of the data-processing steps depend on the details of the experiment and the sequencing technology, but the steps themselves remain the same [6]. Read sequences are cleaned to remove low-quality regions and sequencing adapters before being assembled to give a collection of contigs, or putative transcripts. To gain an insight into the functions of the genes represented by these transcripts, and to identify novel transcripts, the contigs are annotated using a variety of methods. Typically, researchers annotate contigs using a combination of similarity to known sequences and protein domains [7,8] and machine-learning methods which identify features such as transmembrane domains and signal peptides [9,10]. These annotations can be used to put the putative transcripts in biological context [11,12].

### Need for tools

The increase in the availability of transcribed sequence data places corresponding demands on the bioinformatic tools used to make sense of it. While tools have been developed to carry out the tasks of cleaning [13,14], assembling [6,15,16] and annotating [8-10] transcribed sequence data, integration of these tools into a pipeline is generally on an *ad-hoc* basis and in a manner that is not user-friendly. As high-throughput sequencing becomes more pervasive, analysis tools that can be used by biological researchers who are not expert bioinformaticians to both create and investigate annotated transctriptomes will become essential. The increasing volume of sequence data also puts pressure on methods of data dissemination. Publications and raw sequence data resulting from transcriptome sequencing projects are generally made available and archived, but intermediate, detailed annotations are typically not.

### Existing solutions

Some tools exist that partially address these needs, most focussing on either the process of annotation or visualization (Table 1). PartiGene [17] is an integrated pipeline for processing Sanger dideoxy Expressed Sequence Tag (EST) data. It employs a similarity-based assembly process, built for clustering Sanger sequence data, that is not suitable for next-generation sequence data, and the analysis portion of the tool requires considerable technical expertise to use. CBrowse [18] is a recently-published web application that provides an interface to pre-assembled contig and read mapping data. Its focus is on identifying polymorphisms, repeats and sequencing errors rather than on annotation. Many existing annotation tools also have user-friendly web interfaces [8-10,19], but they are generally geared towards small numbers of input sequences and do not integrate multiple types of annotation.

**Table 1:** Comparison of existing software tools designed to work on whole transcriptome datasets

**Name**	**In active development**	**Sequence data type**	**Interface**	**Annotations**
PartiGene [17]	No	Sanger EST reads	Command-line	BLAST, InterProScan, KEGG, prot4est
Cbrowse [18]	No	Assembed contigs + read mapping data	Web	None
BRIGEP [20]	No	Assembled transcripts	Web	BLAST, InterProScan
TranscriptomeBrowser [21]	Yes	Public microarray data	Java GUI	Existing ontologies
afterParty	Yes	Roche 454 raw reads or assembled contigs	Web	BLAST, InterProScan

The scope of this table is restricted to tools designed for transcriptome analysis – genome browsers are not included. We define a project as being in active development if the most recent change to the source code repository was made less than one year ago.

Several tools offer potential solutions for data exploration. BRIGEP [20] is a suite of tools that includes a transcriptome browser to address the need for data visualization. However, BRIGEP is focussed on integration with proteomic data, requires significant technical ability to set up, and does not assist the user in creating annotation. Similarly, the TranscriptomeBrowser [21] tool offers an interface to existing transcript data with a focus on molecular interactions. Genome browsers [22-24] are feature-rich, but they typically require considerable effort to set up, and the gene-centric requirements of transcriptome analysis and visualization do not fit well into their genome- and chromosome-centric paradigm [25,26].

To address the need for an integrated, dependency-free, intuitive tool for transcriptome annotation and publication we have developed afterParty, a web application that runs entirely within a browser and functions both as an annotation tool and a transcriptome browsing and visualization tool. afterParty takes as its input either raw reads or assembled contigs, and uses existing best-practice tools and databases to annotate them, resulting in collections of annotated putative transcripts (“datasets”) along with metadata describing how the sequences were produced. afterParty also acts as a web interface to datasets, allowing non-bioinformatician users to browse contigs, search annotation, and define and visualize sets of contigs. Using afterParty, a biologist can turn a collection of next-generation sequencing reads into a durable, web-accessible transcriptome resource without the need for expert knowledge, software dependencies, or extensive computing power.

## Implementation

The afterParty web application functions as an interface to two sets of tools – one for creating datasets, and one for searching, browsing and visualizing them. To create a new dataset, the user uploads either a set of raw sequencing reads which afterParty assembles into contigs, or a collection of pre-assembled contigs (generated using any appropriate combination of sequencing technology and assembly software) and, optionally, coverage and quality data. Contigs are then annotated and the annotations indexed for rapid searching. To investigate an existing dataset, a user can browse individual contigs or search within datasets for contigs of interest. Searches can interrogate the annotations or contig properties (coverage, GC content, etc.) and can be performed across multiple assemblies in a dataset (e.g. for different species or different RNA libraries). afterParty is implemented as a web application and is written in Groovy [27] using the Grails [28] web framework and the PostgreSQL Relational Database Management Server [29] for data storage. It is offered as a publicly-available server at http://afterparty.bio.ed.ac.uk, but can also be downloaded and run locally.

### Dataset structure

**Figure 1:**
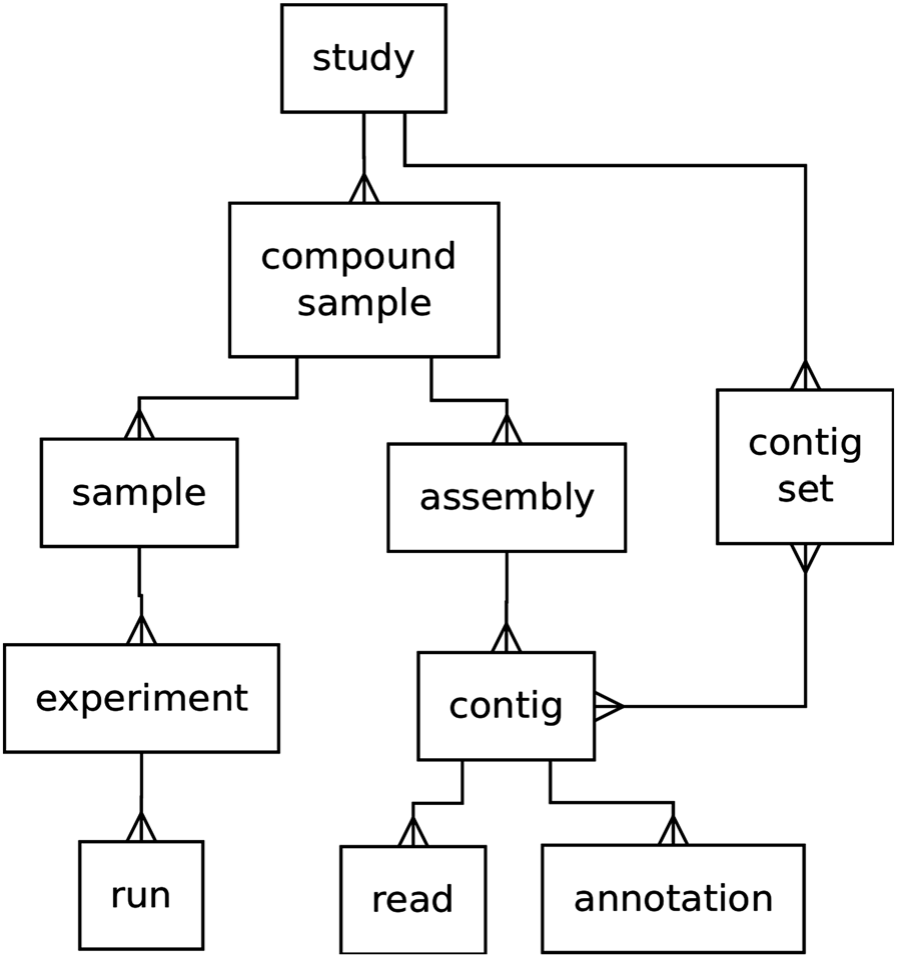
**An entity-relationship diagram of the objects that make up an afterParty dataset.** The structure of relationships is a straightforward one-to-many hierarchy, with the exception of *contigs* and *contig sets* which are in a many-to-many relationship.

afterParty datasets are organized into a structure with two overlapping hierarchies – one for raw sequence data, and one for assembled sequence data (Figure 1). The raw sequence data hierarchy has been designed to be congruent with the The International Nucleotide Sequence Database Collaboration (INSDC) BioProject [30] schema to ease integration with raw sequence archives, and is described here from the bottom-up for clarity. A *run* contains read data for a single sequencing run, and an *experiment* may contain reads from several independent *runs*. Each *experiment* in a dataset may have different RNA preparation and sequencing technologies. *Experiments* are grouped together into *samples*, which reflect separate biological sources of RNA material – for example, different tissue types, life stages, or environmental conditions. A *compound sample* represents a collection of *samples* and usually corresponds to a single species or strain of source organism. Finally, a *study* is a collection of related compound samples, such as a group of closely-related species.

Putative transcripts are represented in afterParty by *contigs,* which are grouped into *assemblies*. A *compound sample* can have multiple *assemblies*. Using this mechanism it is possible to have multiple versions of an assembly for a single set of reads. A *contig* may be decorated with multiple pieces of information, each of which is represented by an *annotation*. Each individual input sequence that makes up a *contig* is represented as a *read*. Arbitrary collections of contigs are stored as *contig sets*. A contig can belong to any number of *contig sets*.

### Adding data

**Figure 2:**
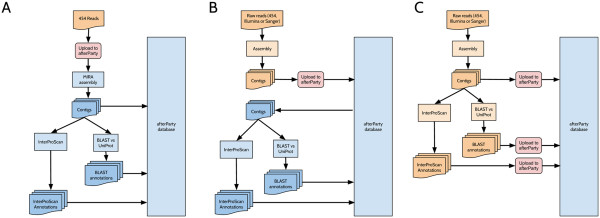
**Flowcharts showing the three possible workflows offered by afterParty.** Orange boxes represent data and processes external to the afterParty server. Blue boxes represent internal afterParty data and processes. Red boxes indicate steps where data are uploaded to afterParty. In workflow **A**, the user uploads raw 454 sequencing reads and both assembly and annotation are carried out inside afterparty. In workflow **B**, assembly is carried out externally. The user uploads a set of assembled contigs, and annotation is carried out inside afterParty. In workflow **C**, both assembly and annotation are carried out externally. The user uploads assembled contigs and their accompanying annotation. While workflow **A** is only suitable for Roche 454 sequencing data and relies on the MIRA assembler, workflows **B** and **C** can be applied to any type of sequencing chemistry or assembly protocol.

afterParty is able to accept input at any stage in the annotation workflow outlined above. Briefly, there are three ways to create a dataset within afterParty (Figure 2). For data derived from 454 pyrosequencing, afterParty can be used for both assembly and annotation (workfow A). For data derived from other sequencing methodologies (e.g. Illumina Solexa RNA-seq) afterParty, assembly must be carried out before data are uploaded to afterParty (workflows B and C).

#### Workflow A: Upload a collection of raw sequencing reads, and allow afterParty to assemble and annotate them

In this scenario, the user uploads a collection of 454 pyrosequencing reads in FASTQ format. afterParty will carry out read assembly using the MIRA assembler [31], optionally trimming adapter sequences using ea-utils [32]. It will then annotate each resulting contig by carrying out a sequence similarity search using BLASTX [19] against the UniProt [7] database of known protein sequences, and running InterProScan [8] to identify known protein domains. Quality and coverage information for each base in each contig as reported by the assembler will be stored along with the contig sequence, annotation, and read mapping locations.

#### Workflow B: Upload a collection of assembled contigs, and allow afterParty to annotate them

In this scenario, the user has already assembled their sequencing reads into contigs and has various choices for uploading them. They can upload a FASTA format file containing contigs, in which case no coverage, quality or read mapping data will be stored, or they can upload an ACE [33] format file which contains coverage, quality and read mapping information. Once uploaded and stored, contigs are annotated as described in workflow A.

Transcriptome assembly from high-throughput data remains an active field of research. Thus workflow B allows users to apply methods best suited to their data type and organism(s) to generate an optimal contig set. In particular, this scenario is likely to be useful for Illumina RNA-Seq sequence data, as well as for complex or large 454 or Sanger transcriptomes that are unlikely to be assembled well by the default Mira assembler. Hybrid approaches to transcriptome assembly, in which output from multiple assembly tools is merged, can also be used under this scenario [15].

#### Workflow C: Upload a collection of assembled contigs along with annotation

In this scenario, the user has already assembled a collection of contigs and run the necessary annotation tools. Contigs are uploaded as described for scenario B, and annotation data are uploaded in either XML (for BLASTX [19]) or GFF3 (for InterProScan [8]) format. No assembly or annotation is carried out by afterParty; the data are merely stored and indexed. This scenario is likely to be useful for users who have access to parallel compute facilities that can carry out the annotation more rapidly than could be accomplished using afterParty. This workflow allows the use of any BLAST database for annotation – for instance, a genome database for a closely-related organism.

In all three workflows datasets remain private, and only visible to the logged-in owner, until explicitly made public.

### Annotation

For the workflows where annotation is carried out inside afterParty (B and C above), annotation proceeds in two steps. First, BLASTX [19] from the BLAST+ 2.2.25 package is used to search the UniProt [7] protein reference database for sequences showing sequence similarity to the contig sequence. The ten most highly similar UniProt entries are stored as annotation, along with their E-value scores and the regions of the contig to which they show similarity. Second, the InterProScan 5 package [8] is used to identify protein domains and regions of interest on the contig using the following applications: *ProDom-2006.1, PfamA-26.0, TIGRFAM-12.0, SMART-6.2, Gene3d-3.3.0, Coils-2.2, Phobius-1.01*[34]. All InterProScan matches are stored along with their E-value scores (where applicable) and positions.

### Browsing, searching and contig sets

Once a dataset has been created, afterParty offers users a variety of ways to explore it. All annotations, whether generated by afterParty or uploaded by the user, are indexed using PostgreSQL's full-text indexing tools. These improve the quality of search results by removing common English words, dealing with suffixes, and allowing boolean search terms. Users can browse a table of the contigs belonging to a particular *assembly*, *compound sample*, or *study*. Alternatively, they can use any of afterParty's search tools to identify contigs of interest. There are three ways to search in afterParty. To search by annotation, users supply a search string (which can include the boolean operators AND, NOT and OR) and afterParty will identify the set of contigs that have matching annotation. To search by similarity, users supply an input DNA or protein sequence and afterParty uses BLASTN, TBLASTN or TBLASTX to carry out a sequence similarity search and identify contigs with significant similarity. To search by contig property (any combination of GC content, read coverage, quality and length), users select a region of a scatter plot encompassing the values they wish to include.

Search results can be saved as *contig sets*, so that they can be retrieved or shared with colleagues without having to re-run the search. Searches can also be restricted to contig sets, leading to a powerful and intuitive way to identify contigs of interest by iteratively combining different types of search. For example, a user can start with a set of contigs from a particular developmental stage, search inside that set for contigs with a particular protein domain, then search inside the resulting set for contigs longer than a minimum length.

### Viewing contig data

**Figure 3:**
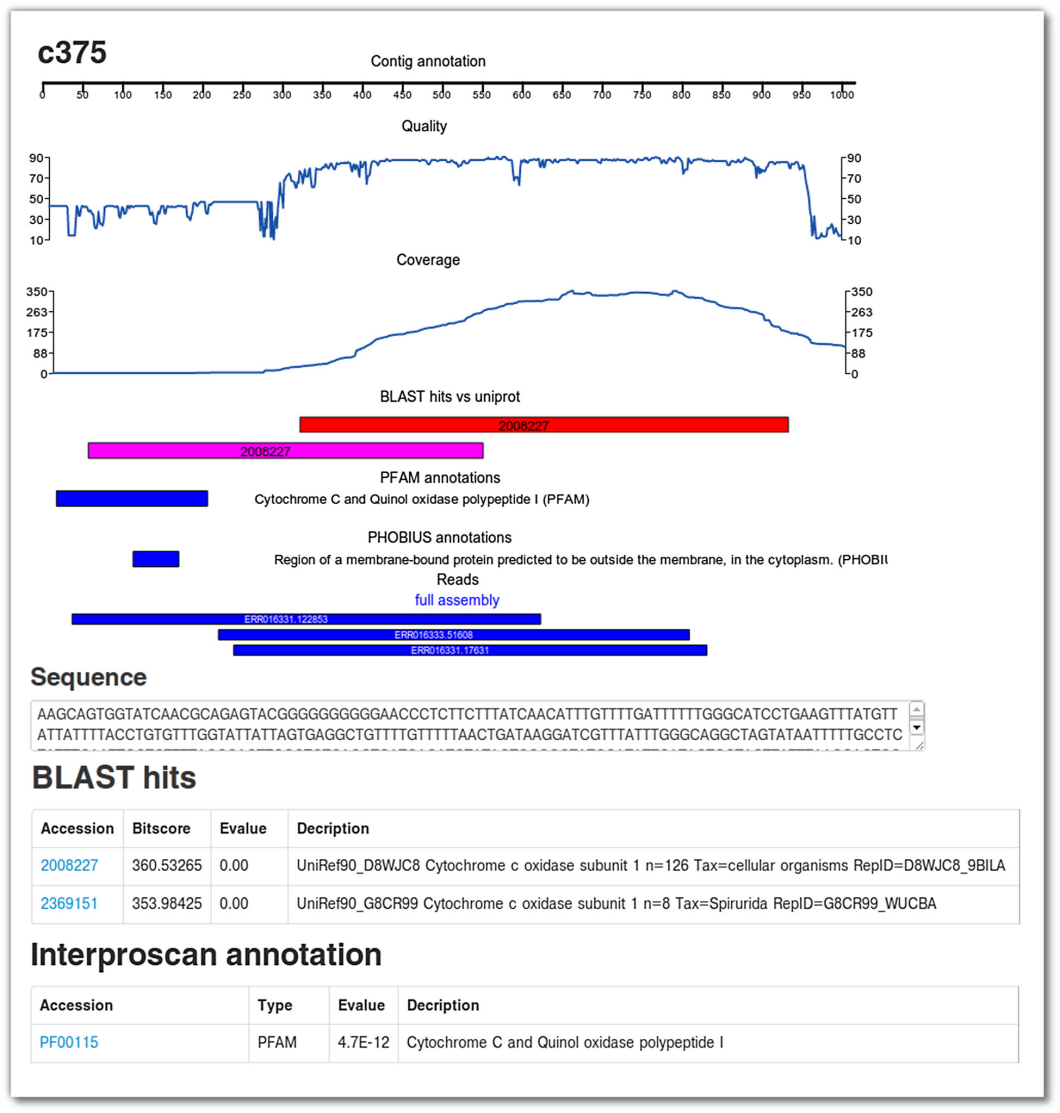
**A screen capture of the user interface for viewing annotation and metadata associated with a single contig taken from the *L. sigmodontis *dataset.** The page contains an overview diagram showing from top to bottom: scale bar; base-by-base coverage and quality score charts; BLAST hits vs. UniProt; InterProScan annotations; read mappings. Below are tables giving details of annotation items and links to relevant external resources. Portions of the interface have been removed from this screen capture in order to fit on the page. The full version of this page can be viewed on the afterParty website [35].

Once contigs of interest have been identified, afterParty allows users to view all the information associated with a particular contig on a single page. Figure 3 shows an example overview page for a contig derived from previously published data [15]. The contig annotation display gives a graphical overview of the annotation and metadata associated with the contig, including charts of quality and read coverage (if available), location and significance of sequence similarity and protein domain annotations, and alignment of sequencing reads. Quality and read coverage will only be available if the assembly was either carried out inside afterParty or uploaded in ACE format. Quality scores are reported by the assembly software and follow the PHRED specification [35]; coverage scores are calculated by afterParty from the positions of the reads. Below the graphical overview are tables listing details of individual annotations, along with links to relevant external resources (known sequences and protein domains).

### Visualization

Grouping of contigs into contig sets allows in depth exploration of properties within and between sets. afterParty automatically creates contig sets for entire assemblies, compound samples, and studies. Database owners and users can define additional contig sets based on particular properties of contig annotation, such as stage-specific expression, or the results of a sequence similarity search.

**Figure 4:**
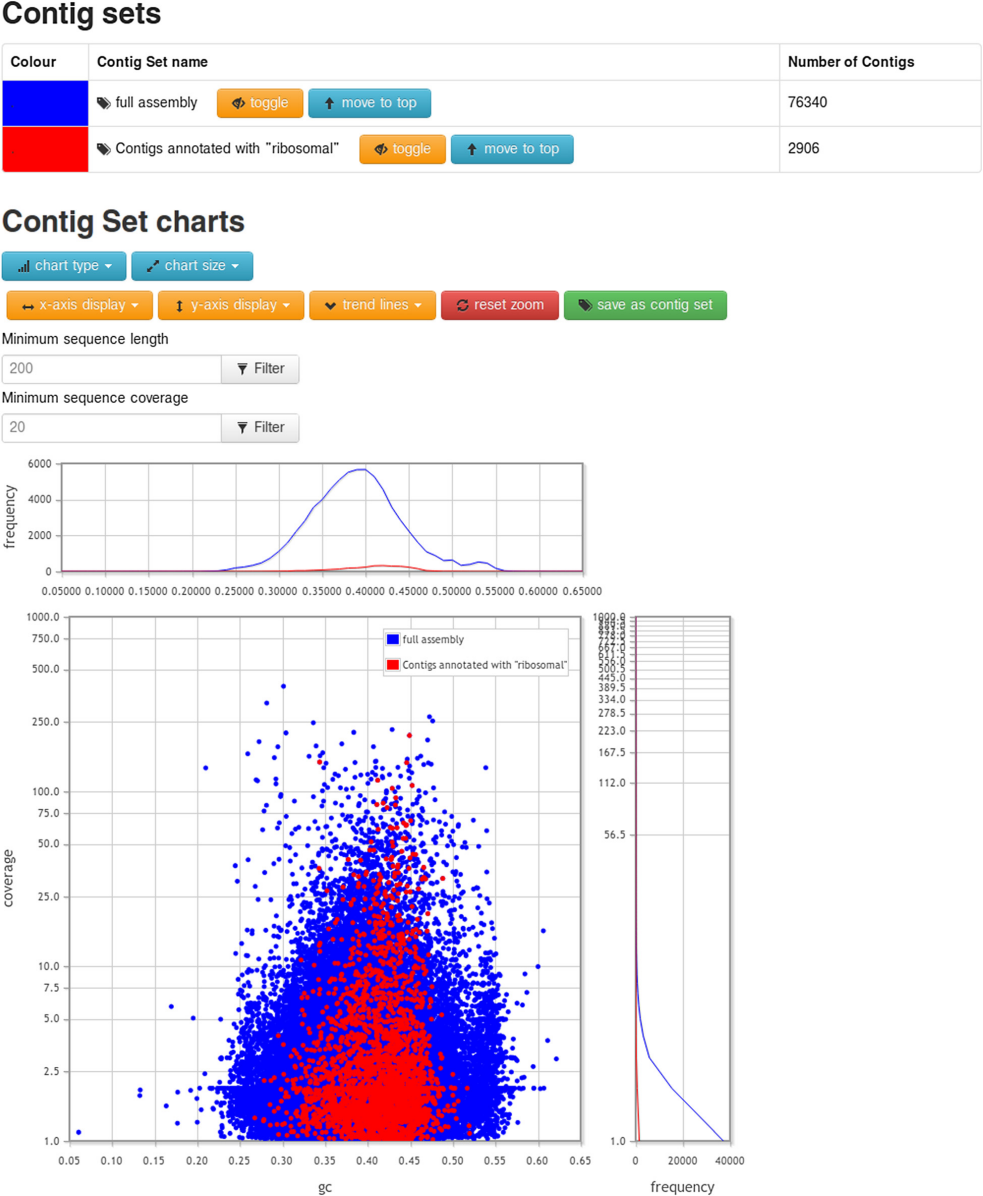
**A screen capture of the user interface for visualizing contig sets.** A user-created contig set containing all contigs with annotation matching the search query “ribosomal” is being compared with the automatically-created contig set containing all contigs for the nematode *Litomosoides sigmodontis.* At the top of the page is a table showing the colour key and contig count for each contig set, along with buttons to toggle their visibility. Below is an area containing a scatter plot of contigs along with a set of chart controls. The scatter plot displays each contig as a single point, coloured according to the key. The x-axis shows GC content and the y-axis shows coverage on a logarithmic scale. Above and to the right of the scatter plot are histograms showing the same data. Clicking on a single point will take the user to an overview for that contig (see Figure 3). Chart controls allow the user to switch between different types of charts; set the axes; filter the displayed contigs; and zoom in on particular regions of the chart. The full, interactive version of this page can be viewed on the afterParty website [36].

To view and compare contig sets, afterParty contains a number of interactive visualization tools (Figure 4). Numerical attributes of contigs (length, length excluding undefined bases, quality, read coverage and GC content) can be displayed either as a scatter plot or a histogram. Scatter plots can have any combination of available axes, which can be linear or logarithmic. Trend lines can be included, and the user can zoom in on any portion of the chart. Hovering over a single point, corresponding to a single contig, displays a pop-up with detailed information about the chosen contig, and clicking takes the user to the overview page for that contig. Users can save contigs that fall within a zoomed region as a new contig set. Histograms can show the distribution of contigs along any single axis, and the frequency axis can be linear or logarithmic. When comparing multiple contig sets, frequencies can be scaled relative to contig set size in order to facilitate comparisons. Both scatter plots and histograms allow the user to exclude very short contigs or those with very low coverage. When comparing multiple contig sets, each is shown on the same axes as a different coloured data series. The user can toggle the visibility of a given contig set, or bring a particular contig set to the top of the chart to ease comparisons.

## Results and discussion

To demonstrate the capabilities of afterParty we used the system to generate publicly available annotated datasets for three transcriptomes from 'neglected' organisms (Table 2).

**Table 2:** Example datasets

**Description**	**URL**	**Number of contigs**	**Number of annotations**	**Data source**	**AfterParty workflow (see Figure**2**)**
Transcriptome of the nematode *Litomosoides sigmodontis* from three life stages	[37]	76,340	770,905	Roche 454 FLX / Titanium	A
Transcriptome of the nematode *Anguilicolla crassus*	[38]	14,064	145,130	Roche 454 FLX / Titanium	B
Transcriptome of the moth *Plodia interpunctella*	[39]	116,191	1,446,916	Illumina Solxa RNA-seq	C

### *Litomosoides sigmodontis* transcriptome

We assembled and annotated a collection of transcriptome sequence data from the filarial nematode *Litomosoides sigmodontis* using the workflow depicted in Figure 2A*. L. sigmodontis* is the subject of an ongoing transcriptome project [15]*,* and the transcriptome data is typical of the type for which we expect afterParty to be useful. 764,024 reads from five libraries were assembled, and annotated using an installation of afterParty on an 8-core server. Assembly took ~48 hours and annotation took ~5 days. The resulting dataset has 76,340 contigs. 69,355 have at least one UniProt annotation, and 24,491 have at least one protein domain annotation. The dataset can be explored on the afterParty web server [37]*.* A subset of these raw *L. sigmodontis* data are available as a test dataset for new users.

### *Anguilicolla crassus* transcriptome

We used a collection of already-assembled transcripts to create a transcriptome resource for the nematode *Anguilicolla crassus* using the workflow depicted in Figure 2B. Sequencing reads for male, female, and L3 individuals were generated using Roche/454 FLX Titanium chemistry and assembled using a hybrid strategy. The assembled contigs were uploaded before being annotated using afterParty. The resulting dataset has 14,064 contigs. 12,625 had at least one UniProt annotation and 6,583 had at least one protein domain annotation. The dataset can be explored on the afterParty web server [38]*. A. crassus* transcriptome assembly data were kindly provided by Emanuel Heitlinger (Berlin) [40].

### *Plodia interpunctella* transcriptome

We used a collection of already-assembled transcripts along with existing annotation to create a transcriptome resource for the Indian Meal Moth, *Plodia interpunctella,* using the workflow depicted in Figure 2C*.* The assembly was built using Trinity [41] from RNA-seq data derived from four samples of fourth instar lavae, each consisting of 20 pooled individuals. Annotation was generated using BLAST [19] and InterProScan [8] on a Sun Grid Engine (SGE) compute cluster. The assembled contigs and annotation files were uploaded to afterParty to create a dataset with 116,191 contigs. 71,608 contigs had at least one UniProt annotation and 16,373 contigs had at least one protein domain annotation. The data can be explored on the afterParty web server [39]*. P. interpunctella* transcriptome assembly data were kindly provided by Seanna McTaggart (University of Edinburgh).

### Assembly and annotation timing

Since afterParty acts as a wrapper around existing third-party tools for assembly and annotation, the overhead imposed versus running the tools manually is minimal. For a test dataset of 100,000 Roche 454 reads take from the *L. sigmodontis dataset*[15]*,* assembly using MIRA [31] took 41 minutes using afterParty compared with 35 minutes when run manually. Annotating a subset of 100 contigs using a BLASTX [19] search vs. UniProt [7] took 407 seconds in afterParty compared with 394 seconds when run manually. Running InterProScan [8] against the same set of 100 contigs took 270 minutes in afterParty compared with 254 minutes when run manually. Timing tests were carried out on a workstation with 4 Intel Xeon L5640 2.27GHz CPUs.

### Development and deployment

We have designed afterParty to be locally deployable for researchers who wish to host datasets themselves, take advantage of local compute facilities, and maintain fine-grained access control. Local deployment of afterParty can be carried out in two ways. The source code is freely available (see Availability and Requirements) and can be installed (along with dependencies) on a standard web server. Alternatively, we have made available a virtual disk image including afterParty and all dependencies, which may be used to create a virtual machine running afterParty. afterParty has been tested using multiple datasets of between ~10,000 and ~250,000 contigs and found to run satisfactorily for dataset browsing and visualization on a 2-core web server with 4 GB RAM.

A single afterParty instance is capable of serving multiple datasets, so we anticipate that a single local installation will be sufficient to serve the needs of a group of researchers working on different projects. The afterParty interface has been designed to facilitate collaboration and sharing of information and is designed such that each study, compound sample, assembly, contig set and contig has a unique URL. Users can easily share a link to a given resource by embedding the URL in an email or web page.

An entire afterParty instance (potentially containing many datasets) can be archived either as a database dump or as a virtual disk image. Database dumps are more compact and hence easier to store. However, recent long-term archival solutions achieve storage costs on the order of $0.01 per gigabyte per month [42]*,* making the storage of complete virtual machines a realistic option (we estimate the size of a complete VM image for a large afterParty instance to be less than 20GB).

### Outlook

We anticipate that the need for tools like afterParty will increase as next-generation sequencing technologies become ever more accessible. In particular, we see a role for afterParty in presenting transcriptome studies which encompass multiple related organisms, aggregating data across research projects.

Obvious extensions to afterParty are the inclusion of additional assembly options and of new types of annotation data. Although the MIRA assembly tool has been shown to produce suboptimal assemblies for some datasets [15], we chose it for use in afterParty because of its modest computational requirements, non-restrictive license, and ease of integration. We plan to integrate additional assembly tools and strategies into afterParty, which will allow the use of input data from other sequencing platforms. The modular design of afterParty's annotation framework ensures that new types can be easily added. We plan to add storage for expression data, such as microarray data and sequence-counting estimates of transcript abundance, open reading frames, matches to proteomics resources, and pathway annotations. We believe that the use of cross-species contig sets to store ortholog relationships will be particularly useful. We also plan to add export tools to afterParty that will aid users in preparing data for submission to annotation archives, such as the International Nucleotide Sequence Database Collaboration (INSDC) Third Party Annotation (TPA) databases.

The computational requirements of afterParty vary throughout the workflow in a distinctive way. The assembly stage can have high memory requirements, and the annotation stage can have high CPU requirements. Once a dataset has been assembled and annotated, however, the memory and CPU resources needed to serve it are modest. CPU-intensive operations such as searching annotations are very brief (in tests, our web server [2 CPU cores @ 2.50 GHz] was able to carry out a full-text search on a dataset with 1.2 million annotation items in under a second). This pattern of transient high demand (during assembly and annotation) and long-term low demand (during browsing and searching) makes afterParty a good candidate for cloud-based compute infrastructure. We are currently investigating the possibility of implementing a highly parallel cloud computing model for the afterParty annotation pipeline.

## Conclusions

afterParty is an open-source tool for turning raw transcriptome sequencing reads and assembled contigs into searchable, browsable transcriptome resources with powerful visualization tools. In contrast to existing solutions, afterParty integrates all steps of the transcriptome annotation workflow and presents an intuitive user interface for non-expert users, while being flexible enough to accommodate assemblies and annotations produced by more experienced users. It implements best-practise assembly and annotation methods, and facilitates data sharing and visualization. It is our hope that, by easing the process of annotation, publication, and stable archiving, afterParty will facilitate the distribution and exploration of richly-decorated transcriptome data that would otherwise remain inaccessible.

## Availability and requirements

**Project name**: afterParty

**Project home page**: https://github.com/mojones/afterParty2

**Operating system**: platform independent (developed on Ubuntu Linux 12.04)

**Programming language**: Groovy [http://groovy.codehaus.org/]

**Other requirements**:

Git [http://git-scm.com/]

Java  1.6  [http://www.oracle.com/technetwork/java/javase/downloads/index.html]

Grails 2.0.3 [http://grails.org/]

**Grails plugins**:

Executor [http://www.grails.org/plugin/executor]

Spring security [http://grails.org/plugin/spring-security-core]

Spring  security  UI  [http://grails.org/plugin/spring-security-ui]

PostgreSQL 9.1 [http://www.postgresql.org/]

NCBI blast+ 2.2.25 [ftp://ftp.ncbi.nlm.nih.gov/blast/executables/blast+/LATEST/]

UniProt [http://www.uniprot.org/downloads]

InterProScan 5 [http://code.google.com/p/interproscan/]

Mira 3.2.1 [http://sourceforge.net/projects/mira-assembler/]

**License**: GNU GPL

**Any restrictions to use by non-academics**: no

### Availability notes

Because of the number of dependencies that afterParty relies on, we have made the software available in three different ways.

### Via the public server at afterParty.bio.ed.ac.uk

No special credentials are necessary to browse published datasets. We are happy to host new transcriptome datasets on this server; please contact the corresponding author (MJ) to obtain a user account. To get started, follow the various tutorials either on the wiki [https://github.com/mojones/afterParty2/wiki/afterParty], or as screencasts [http://www.youtube.com/user/theblaxterlab/videos].

### By downloading the source code and installing dependencies

The source code for afterParty is hosted at GitHub [https://github.com/mojones/afterParty2]. Pull requests are welcome. Bugs and feature requests can also be submitted at the above address. Follow the installation instructions here: https://github.com/mojones/AfterParty2/wiki/LocalInstall.

### By downloading a virtual disk image

To assist researchers who would like to run a local installation of afterParty, we have prepared a virtual disk image, based on Ubuntu (server) 12.04, which can be run under a virtual machine hypervisor such as VirtualBox. The virtual disk image expands to around 80 GB and requires a 64-bit host. This is the easiest way to get afterParty running locally as all necessary dependencies and permissions are already set up. Follow the installation instructions here: https://github.com/mojones/AfterParty2/wiki/VMInstall.

## Competing interests

The authors declare that they have no competing interests.

## Authors’ contributions

The project was conceived by MJ and MB. The software was designed by MJ and MB and implemented by MJ. The *L. sigmodontis* afterParty dataset was assembled by MJ. Both authors read and approved the final manuscript.

## References

[B1] Shendure J Ji H Next-generation DNA sequencing Nat Biotechnol 2008 26 1135 1145 18846087 10.1038/nbt1486

[B2] Abecasis GR Altshuler D Auton A Brooks LD Durbin RM Gibbs RA Hurles ME McVean GA A map of human genome variation from population-scale sequencing Nature 2010 467 1061 1073 20981092 10.1038/nature09534 PMC3042601

[B3] Graveley BR Brooks AN Carlson JW Duff MO Landolin JM Yang L Artieri CG Baren MJ Van Boley N Booth BW Brown JB Cherbas L Davis CA Dobin A Li R Lin W Malone JH Mattiuzzo NR Miller D Sturgill D Tuch BB Zaleski C Zhang D Blanchette M Dudoit S Eads B Green RE Hammonds A Jiang L Kapranov P Langton L The developmental transcriptome of Drosophila melanogaster Nature 2011 471 473 479 21179090 10.1038/nature09715 PMC3075879

[B4] Feldmeyer B Wheat CW Krezdorn N Rotter B Pfenninger M Short read Illumina data for the de novo assembly of a non-model snail species transcriptome (Radix balthica, Basommatophora, Pulmonata), and a comparison of assembler performance BMC Genomics 2011 12 317 21679424 10.1186/1471-2164-12-317 PMC3128070

[B5] Parchman TL Geist KS Grahnen JA Benkman CW Buerkle CA Transcriptome sequencing in an ecologically important tree species: assembly, annotation, and marker discovery BMC Genomics 2010 11 180 20233449 10.1186/1471-2164-11-180 PMC2851599

[B6] Martin JA Wang Z Next-generation transcriptome assembly Nat Rev Genet 2011 12 671 682 21897427 10.1038/nrg3068

[B7] Reorganizing the protein space at the Universal Protein Resource (UniProt) Nucleic Acids Res 2012 40 71 75 10.1093/nar/gkr981 PMC3245120 22102590

[B8] Zdobnov EM Apweiler R InterProScan–an integration platform for the signature-recognition methods in InterPro Bioinformatics 2001 17 847 848 11590104 10.1093/bioinformatics/17.9.847

[B9] Sonnhammer EL von Heijne G Krogh A A hidden Markov model for predicting transmembrane helices in protein sequences Proc Int Conf Intell Syst Mol Biol 1998 6 175 182 9783223

[B10] Nielsen H Brunak S von Heijne G Machine learning approaches for the prediction of signal peptides and other protein sorting signals Protein Eng 1999 12 3 9 10065704 10.1093/protein/12.1.3

[B11] Ashburner M Ball CA Blake JA Botstein D Butler H Cherry JM Davis AP Dolinski K Dwight SS Eppig JT Harris MA Hill DP Issel-Tarver L Kasarskis A Lewis S Matese JC Richardson JE Ringwald M Rubin GM Sherlock G Gene ontology: tool for the unification of biology. The Gene Ontology Consortium Nat Genet 2000 25 25 29 10802651 10.1038/75556 PMC3037419

[B12] Kanehisa M Goto S Sato Y Furumichi M Tanabe M KEGG for integration and interpretation of large-scale molecular data sets Nucleic Acids Res 2012 40 D109 114 22080510 10.1093/nar/gkr988 PMC3245020

[B13] Kong Y Btrim: a fast, lightweight adapter and quality trimming program for next-generation sequencing technologies Genomics 2011 98 152 153 21651976 10.1016/j.ygeno.2011.05.009

[B14] Lindgreen S AdapterRemoval: easy cleaning of next-generation sequencing reads BMC Res Notes 2012 5 337 22748135 10.1186/1756-0500-5-337 PMC3532080

[B15] Kumar S Blaxter ML Comparing de novo assemblers for 454 transcriptome data BMC Genomics 2010 11 571 20950480 10.1186/1471-2164-11-571 PMC3091720

[B16] Mundry M Bornberg-Bauer E Sammeth M Feulner PGD Evaluating Characteristics of De Novo Assembly Software on 454 Transcriptome Data: A Simulation Approach PLoS ONE 2012 7 e31410 22384018 10.1371/journal.pone.0031410 PMC3288049

[B17] Parkinson J Anthony A Wasmuth J Schmid R Hedley A Blaxter M PartiGene–constructing partial genomes Bioinformatics 2004 20 1398 1404 14988115 10.1093/bioinformatics/bth101

[B18] Li P Ji G Dong M Schmidt E Lenox D Chen L Liu Q Liu L Zhang J Liang C CBrowse: a SAM/BAM-based contig browser for transcriptome assembly visualization and analysis Bioinformatics 2012 28 2382 2384 22789590 10.1093/bioinformatics/bts443 PMC3436847

[B19] Altschul SF Madden TL Schäffer AA Zhang J Zhang Z Miller W Lipman DJ Gapped BLAST and PSI-BLAST: a new generation of protein database search programs Nucleic Acids Res 1997 25 3389 3402 9254694 10.1093/nar/25.17.3389 PMC146917

[B20] Goesmann A Linke B Bartels D Dondrup M Krause L Neuweger H Oehm S Paczian T Wilke A Meyer F BRIGEP–the BRIDGE-based genome-transcriptome-proteome browser Nucleic Acids Res 2005 33 W710 716 15980569 10.1093/nar/gki400 PMC1160161

[B21] Lepoivre C Bergon A Lopez F Perumal NB Nguyen C Imbert J Puthier D TranscriptomeBrowser 3.0: introducing a new compendium of molecular interactions and a new visualization tool for the study of gene regulatory networks BMC Bioinformatics 2012 13 19 22292669 10.1186/1471-2105-13-19 PMC3395838

[B22] Kent WJ Sugnet CW Furey TS Roskin KM Pringle TH Zahler AM Haussler D The human genome browser at UCSC Genome Res 2002 12 996 1006 12045153 10.1101/gr.229102 PMC186604

[B23] Skinner ME Uzilov AV Stein LD Mungall CJ Holmes IH JBrowse: a next-generation genome browser Genome Res 2009 19 1630 1638 19570905 10.1101/gr.094607.109 PMC2752129

[B24] Stein LD Mungall C Shu S Caudy M Mangone M Day A Nickerson E Stajich JE Harris TW Arva A Lewis S The generic genome browser: a building block for a model organism system database Genome Res 2002 12 1599 1610 12368253 10.1101/gr.403602 PMC187535

[B25] Bouétard A Noirot C Besnard A-L Bouchez O Choisne D Robe E Klopp C Lagadic L Coutellec M-A Pyrosequencing-based transcriptomic resources in the pond snail Lymnaea stagnalis, with a focus on genes involved in molecular response to diquat-induced stress Ecotoxicology 2012 21 2222 2234 22814884 10.1007/s10646-012-0977-1

[B26] Papanicolaou A Gebauer-Jung S Blaxter ML Owen McMillan W Jiggins CD ButterflyBase: a platform for lepidopteran genomics Nucleic Acids Res 2008 36 D582 D587 17933781 10.1093/nar/gkm853 PMC2238913

[B27] Groovy - Homehttp://groovy.codehaus.org/

[B28] Grails - The search is overhttp://grails.org/

[B29] PostgreSQL: The world’s most advanced open source databasehttp://www.postgresql.org/

[B30] Karsch-Mizrachi I Nakamura Y Cochrane G The International Nucleotide Sequence Database Collaboration Nucleic Acids Res 2012 40 D33 D37 22080546 10.1093/nar/gkr1006 PMC3244996

[B31] Chevreux B Pfisterer T Drescher B Driesel AJ Müller WEG Wetter T Suhai S Using the miraEST assembler for reliable and automated mRNA transcript assembly and SNP detection in sequenced ESTs Genome Res 2004 14 1147 1159 15140833 10.1101/gr.1917404 PMC419793

[B32] ea-utils - FASTQ processing utilities - Google Project Hostinghttp://code.google.com/p/ea-utils/

[B33] contigimage - create contig images based on .ace filehttp://www.animalgenome.org/bioinfo/resources/manuals/contigimage.html

[B34] Käll L Krogh A Sonnhammer ELL A combined transmembrane topology and signal peptide prediction method J Mol Biol 2004 338 1027 1036 15111065 10.1016/j.jmb.2004.03.016

[B35] Ewing B Green P Base-calling of automated sequencer traces using phred. II. Error probabilities Genome Res 1998 8 186 194 9521922

[B36] Contig set comparison http://afterparty.bio.ed.ac.uk/contigSet/compareContigSets?check_669824=on&check_1440737=on

[B37] Study | 454 Sequencing of Litomosoides sigmodontis transcriptome from 3 lifestageshttp://afterparty.bio.ed.ac.uk/study/show/5

[B38] Study | Transcriptome of the nematode Anguilicolla crassushttp://afterparty.bio.ed.ac.uk/study/show/1440745

[B39] Study | De novo transcriptome assembly of the grain-eating pest, Plodia interpunctella and its natural viral pathogen Plodia interpunctella granulosis virushttp://afterparty.bio.ed.ac.uk/study/show/2194070

[B40] Heitlinger E Bridgett S Montazam A Taraschewski H Blaxter M The transcriptome of the invasive eel swimbladder nematode parasite Anguillicola crassus BMC Genomics 2013 14 87 23394720 10.1186/1471-2164-14-87 PMC3630068

[B41] Grabherr MG Haas BJ Yassour M Levin JZ Thompson DA Amit I Adiconis X Fan L Raychowdhury R Zeng Q Chen Z Mauceli E Hacohen N Gnirke A Rhind N di Palma F Birren BW Nusbaum C Lindblad-Toh K Friedman N Regev A Full-length transcriptome assembly from RNA-Seq data without a reference genome Nat Biotechnol 2011 29 644 652 21572440 10.1038/nbt.1883 PMC3571712

[B42] Amazon Glacierhttp://aws.amazon.com/glacier/

